# Activation of Myocardial Phosphoinositide-3-Kinase p110α Ameliorates Cardiac Dysfunction and Improves Survival in Polymicrobial Sepsis

**DOI:** 10.1371/journal.pone.0044712

**Published:** 2012-09-19

**Authors:** Chuanfu Li, Fang Hua, Tuanzhu Ha, Krishna Singh, Chen Lu, John Kalbfleisch, Kevin F. Breuel, Tiffany Ford, Race L. Kao, Ming Gao, Tammy R. Ozment, David L. Williams

**Affiliations:** 1 Department of Surgery, Quillen College of Medicine, East Tennessee State University, Johnson City, Tennessee, United States of America; 2 Department of Emergency Medicine, Emory University School of Medicine Atlanta, Georgia, United States of America; 3 Department of Biomedical Sciences, Quillen College of Medicine, East Tennessee State University, Johnson City, Tennessee, United States of America; 4 Department of Biometry and Medical Computing, Quillen College of Medicine, East Tennessee State University, Johnson City, Tennessee, United States of America; 5 Department of Obstetrics and Gynecology, Quillen College of Medicine, East Tennessee State University, Johnson City, Tennessee, United States of America; University of Cincinnati, United States of America

## Abstract

Phosphoinositide-3-kinase (PI3K)/Akt dependent signaling has been shown to improve outcome in sepsis/septic shock. There is also ample evidence that PI3K/Akt dependent signaling plays a crucial role in maintaining normal cardiac function. We hypothesized that PI3K/Akt signaling may ameliorate septic shock by attenuating sepsis-induced cardiac dysfunction. Cardiac function and survival were evaluated in transgenic mice with cardiac myocyte specific expression of constitutively active PI3K isoform, p110α (caPI3K Tg). caPI3K Tg and wild type (WT) mice were subjected to cecal ligation/puncture (CLP) induced sepsis. Wild type CLP mice showed dramatic cardiac dysfunction at 6 hrs. Septic cardiomyopathy was significantly attenuated in caPI3K CLP mice. The time to 100% mortality was 46 hrs in WT CLP mice. In contrast, 80% of the caPI3K mice survived at 46 hrs after CLP (p<0.01) and 50% survived >30 days (p<0.01). Cardiac caPI3K expression prevented expression of an inflammatory phenotype in CLP sepsis. Organ neutrophil infiltration and lung apoptosis were also effectively inhibited by cardiac PI3k p110α expression. Cardiac high mobility group box–1 (HMGB-1) translocation was also inhibited by caPI3K p110α expression. We conclude that cardiac specific activation of PI3k/Akt dependent signaling can significantly modify the morbidity and mortality associated with sepsis. Our data also indicate that myocardial function/dysfunction plays a prominent role in the pathogenesis of sepsis and that maintenance of cardiac function during sepsis is essential. Finally, these data suggest that modulation of the PI3K/p110α signaling pathway may be beneficial in the prevention and/or management of septic cardiomyopathy and septic shock.

## Introduction

The critically ill patient frequently develops a complex disease spectrum that may include acute respiratory distress syndrome (ARDS), systemic inflammatory response syndrome (SIRS), sepsis syndrome and/or septic shock and multi-organ dysfunction syndrome (MODS) [1]. In the United States ∼750,000 patients/year develop sepsis syndrome [2]. The overall mortality rate is 28.6% (∼215,000 deaths/year) [2]. Those patients that survive the initial event may ultimately succumb to widespread organ dysfunction that can be either acute, due to hyper-inflammatory responses, or more prolonged due to immune dysfunction and infection [3], [4]. Indeed, sepsis is a frequent cause of MODS [3], [4]. In many cases, the lung is the primary organ affected, however, it is well known that cardiovascular dysfunction is also associated with MODS morbidity and mortality [5]–[7]. Cardiovascular dysfunction as a consequence of sepsis has been termed “septic cardiomyopathy” [5]–[7]. It is now well accepted that septic cardiomyopathy is an emerging problem in the management of the critically ill patient [5].

The phosphoinositide-3-kinases (PI3Ks) are a conserved family of signal transduction enzymes which are involved in regulating cellular proliferation and survival [8], [9]. The PI3Ks and the downstream serine/threonine kinase Akt (also known as protein kinase B = PKB) regulate cellular activation, inflammatory responses, chemotaxis and apoptosis [9]. Guha and Mackman have reported that the “PI3K-Akt pathway imposes a braking mechanism to limit the expression” of pro-inflammatory mediators in LPS treated monocytes [10]. Using an *in vivo* sepsis model, we have reported that PI3K inhibition increases susceptibility of mice to polymicrobial sepsis [11]. Of greater significance, we have demonstrated that stimulation of PI3K/Akt dependent signaling increases survival outcome in sepsis [11]. In support of this observation, Bommhardt et al reported that mice which constitutively over express active Akt in their lymphocytes showed decreased lymphocyte apoptosis, a T_H_1 cytokine propensity, and a marked improvement in survival outcome in response to sepsis [12]. We and others have reviewed the role of PI3K in the regulation of inflammatory and/or septic responses [13], [14]. The data suggest that PI3K/Akt may be an endogenous negative feedback regulator and/or compensatory mechanism which is crucial to the maintenance and integrity of homeostasis during septic and/or inflammatory insults [13], [14]. However, the mechanisms by which PI3K/Akt ameliorates septic sequelae and improves survival outcome have not been fully delineated.

There is ample evidence that PI3k/Akt dependent signaling plays a crucial role in cardiac function [15]–[17]. We have reported that sepsis decreases myocardial Akt activation [18], which positively correlates with cardiac dysfunction in sepsis. We also reported that preventing sepsis induced changes in Akt activation ameliorates cardiovascular dysfunction in sepsis [18]. Based on these data, we hypothesized that activation of PI3K/Akt dependent signaling will prevent or blunt cardiac dysfunction during sepsis/septic shock. To test this hypothesis, we examined the effect of sepsis/septic shock on cardiac function and survival in transgenic mice with cardiac myocyte specific expression of a constitutively active PI3K isoform p110α (caPI3K Tg).

## Materials and Methods

### Experimental Animals

The caPI3K p110α transgenic mice, on the C47Bl/6J background, were kindly provided by Dr. Seigo Izumo from Harvard Medical School [19], [20]. The mice were maintained and bred in the Division of Laboratory Animal Resources at East Tennessee State University (ETSU). The experiments outlined in this manuscript conform with the Guide for the Care and Use of Laboratory Animals published by the US National Institutes of Health (NIH Publication No. 85–23, revised 1996). The experimental protocols as well as all aspects of animal care described in this manuscript were reviewed and approved by the ETSU Committee on Animal Care.

### Cecal Ligation and Puncture (CLP)

Cecal ligation and puncture was performed to induce sepsis in mice as previously described [21]–[23]. Briefly, the mice were anesthetized by isoflurane inhalation and ventilated with room air using a rodent ventilator. A midline incision was made on the anterior abdomen and the cecum was exposed and ligated with a 0 suture. Two punctures were made through the cecum with an 18-gauge needle and feces were extruded from the holes. The abdomen was then closed. Fluid resuscitation (1 ml lactated Ringers) was administered subcutaneously after surgery. Sham surgical operated mice (anesthesia and laparotomy) served as the surgical control group. Mice that were not subjected to surgery or anesthesia served as the normal controls.

### Experimental Protocol

Mice were subjected to either CLP or sham surgery at time 0. Cardiovascular function was assessed at 6 hrs post-CLP. In a separate experiment, the same groups were followed for survival and at 16 hrs representative post-CLP mice were taken from each group, euthanized and serum and tissues were harvested for analysis of serum cytokines/chemokines, Akt and glycogen synthase kinase-3β (GSK3β) phosphorylation, tissue apoptosis and tissue nuclear factor-кB (NFкB) analysis. For HMGB-1 analysis, tissue samples were harvested at 6 and 12 hrs after CLP.

### Hemodynamic Measurements

Cardiovascular function was assessed as previously described by our group [18]. Briefly, mice were anesthetized with isoflurane inhalation and ventilated with room air using a rodent ventilator. A microconductance pressure catheter (Millar Instruments Inc., Houston, TX) was positioned in the left ventricle (LV) via the right carotid artery for continuous registration of LV pressure-volume loops [24], [25] using the PowerLab system (AD Instruments, Inc., Colorado Springs, CO). A cuvette calibration method was used to convert the conductance voltage into volume units by filling nonconductive cuvettes of known diameter with heparin treated mouse blood. Parallel conductance from surrounding structures was determined by intravenous (external jugular vein) injection of a small bolus (15 ml) of hypertonic saline (15% NaCl). All measurements were performed while ventilation was turned off momentarily. Indices of systolic and diastolic cardiac performance were derived from LV pressure-volume data obtained at steady state.

### Echocardiographic Analysis of Cardiac Function

Transthoracic two-dimensional M-mode echocardiograms were obtained using a Toshiba Aplio 80 Imaging System (Tochigi, Japan) equipped with a 12 MHz linear transducer. The mice were anesthetized using a mixture of isoflurane (1.5%) and oxygen (0.5 l/min) and the body temperature was maintained at ∼37°C using a heating pad. M-mode tracings were used to measure end-diastolic diameter (LVEDD). Percent fractional shortening (%FS) and ejection fraction (%EF) were calculated as described [26]. All echocardiographic assessments were performed by the same investigator.

### Western Blot

Cytoplasmic proteins were isolated from heart, liver, lung and spleen. Immunoblots were performed as described previously [27]–[31]. Briefly, the cellular proteins were separated by sodium dodecyl sulfate (SDS)-polyacrylamide gel electrophoresis and transferred onto Hybond ECL membranes (Amersham Pharmacia, Piscataway, NJ). The ECL membranes were incubated with appropriate primary antibody against anti-phospho-Akt, or anti-Akt; anti-phospho-GSK3β or anti-GSK3β respectively, followed by incubation with peroxidase-conjugated second antibodies (Cell Signaling Technology, Inc. Beverly, MA). The membranes were analyzed by the ECL system (Amersham Pharmacia). The same membranes were stripped and re-probed with anti-GAPDH (glyceraldehyde-3-phosphate dehydrogenase, Biodesign, Saco, Maine) as loading controls. In the case of nuclear HMGB-1, histone 3 was employed as the loading control. The signals were quantified by scanning densitometry and computer-assisted image analysis.

### Assessment of Serum Cytokine Levels

Four to six mice from each group were euthanized at 16 hrs after surgery and the serum was harvested. The serum was stored in liquid nitrogen until assayed. Serum cytokine levels were assayed with an Invitrogen murine 20 plex cytokine assay (Carlsbad, CA) on a Luminex 100 instrument. Specifically, we assayed the serum for fibroblast growth factor basic (FGFb), granulocyte macrophage colony stimulating factor (GM-CSF), interferon-γ (IFN-γ), interleukin-1α (IL-1α), interleukin-1β (IL-1β), interleukin-2 (IL-2), interleukin-4 (IL-4), interleukin-5 (IL-5), interleukin-6 (IL-6), interleukin-10 (IL-10), interleukin-12 p40/p70 (IL-12p40/p70), interleukin-13 (IL-13), interleukin-17 (IL-17), interferon inducible protein-10 (IP-10), keratinocyte chemoattractant (KC), monocyte chemotactic protein-1 (MCP-1), macrophage inflammatory protein-1α (MIP-1α), monokine induced by interferon-γ (MIG), tumor necrosis factor α (TNFα) and vascular endothelial growth factor (VEGF). Cytokine levels were established by comparison to a standard curve as per the manufacturer’s instructions.

### Splenocyte Apoptosis

We employed two approaches to assess splenocyte apoptosis in response to sepsis. First, *in situ* cardiac splenocyte apoptosis was examined by the TdT mediated dUTP nick end labeling (TUNEL) assay (Boehringer Mannheim, Indianapolis, IN) as described previously [24]. Three slides from each block were evaluated for apoptotic cells using the TUNEL assay. Tunel stains were imaged using a fluorescent microscope. In addition, four slide fields were randomly examined using a defined rectangular field area with 200X magnification. Apoptotic cells were counted in each field. Second, spleens were harvested into ice cold phosphate buffered saline (PBS) 16 hrs after induction of CLP. Individual cells were isolated by teasing the stroma apart with needles, allowing the stroma to settle, and pouring the suspended cells into a fresh tube. The red blood cells were lysed with hypotonic saline. The leukocytes were fixed with ice cold 70% ethanol for at least 18 h at −20°C. The cells were washed three times with PBS, and then stained with 50 µg/ml propidium iodide with 10 µg/ml RNase A for 30 min at 37°C. The samples were then analyzed for the sub G0 population using a FACScalibur flow cytometer with CellQuest software (BD Biosciences, Mountain View, CA).

### Tissue Myeloperoxidase (MPO) Activity

Lung tissue was processed as directed by the MPO Fluorometric Detection Kit (Assay Designs, Ann Arbor, MI). Specifically, 50 mg of tissue was weighed out into 1X assay buffer containing 10 mM N-ethylmaleimide (Sigma). The tissue samples were homogenized with a Polytron. After pelleting, the cells were lysed using 0.5% hexadecyltrimethylammonium in 1X assay buffer. Following homogenization and sonication at 50% power for 3–10 s pulses, the homogenates were subjected to two freeze thaw cycles. After clearing of cell debris by centrifugation, the lysates were stored at −80°C until assayed. The samples were assayed according to kit directions and were read using the Modulus Microplate fluorescent plate reader after 30 min incubation (Turner Biosystems, Sunnyvale, CA).

### Tissue NFκB Assay

Nuclear protein was isolated from lung tissues according to previously published methods [21]. Nuclear protein was assayed for NFкB activity using a commercially available kit (Pierce Thermo Scientific, Rockford, IL) according to the manufacturer’s instructions.

### Statistics

Survival trends were compared with the log-rank Wilcoxon non-parametric procedures. Phosphorylation, caspase and cytokine levels were summarized by the group mean. Using replicate averages for each animal, group means were statistically compared with the one-way analysis of variance (ANOVA) followed by the least significant difference test. Unless otherwise stated data are expressed as mean + s.e.m. A probability level of 0.05 or smaller was used to report statistical significance.

## Results

### Cardiac Specific Expression of Constitutively Active PI3K p110α Attenuates Sepsis Induced Cardiac Dysfunction

**Figure 1:**
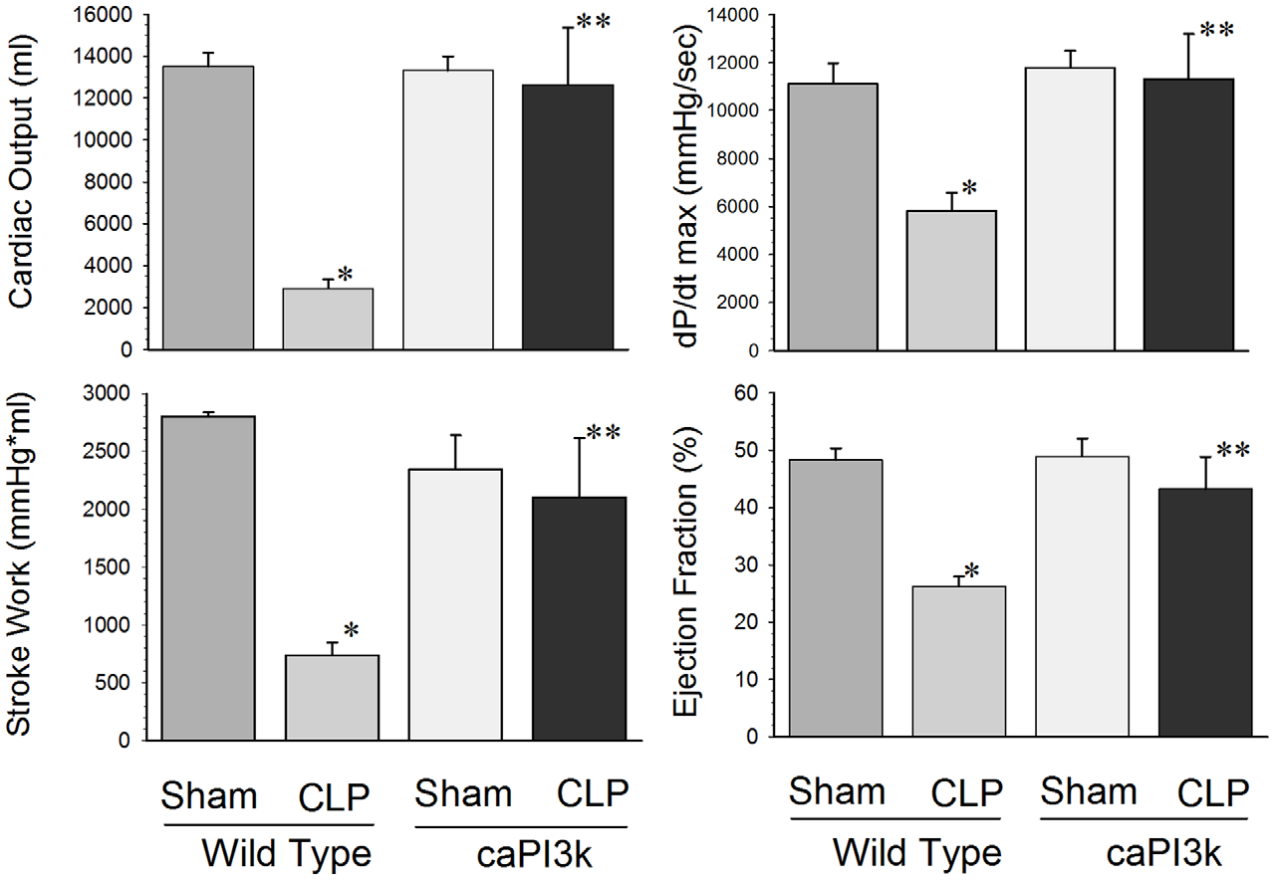
Cardiac myocyte specific expression of PI3K p110α attenuated cardiac dysfunction in CLP-induced septic mice. We performed hemodynamic and echocardiographic analysis at 6 hrs after CLP. Echocardiography confirmed that cardiac function was compromised in WT CLP. In contrast, caPI3K mice with CLP induced sepsis showed an overall maintenance of cardiac function. The bar graphs show the cumulative cardiac function data for the respective control and experimental groups. Six hrs after CLP, left ventricular hemodynamic parameters were examined. N = 5 mice/group. * p<0.05 compared to age-matched WT control. **p<0.05 compared to WT CLP group.

We examined the effect of sepsis on cardiac function in caPI3K mice using two approaches. First, we examined cardiac function using a conductance catheter as previously described [24], [25], [18]. Second, we employed echocardiography as a non-invasive approach to assess cardiac function in septic mice. Cardiac function was assessed at 6 hrs after CLP induced sepsis. As expected, WT mice subjected to CLP showed significant depression of cardiac function (**Table 1** and **Figure 1**). Cardiac output was reduced by 78.5%, stroke work by 73.7%, dp/dt max (mmHg/sec) by 47.6%, and ejection fraction by 45.7%, respectively, compared with sham control (**Figure 1**). However, cardiac dysfunction was attenuated in response to sepsis in mice with cardiac specific expression of constitutively active PI3K p110α, (**Table 1** and **Figure 1**). In some cases, expression of the PI3k p110α transgene resulted in a maintenance of normal cardiac function in response to sepsis (**Figure 1**). Specific examples include cardiac output, dP/dt max, stroke work, ejection fraction, maximum elastance (Emax) and regression of log (pressure) versus time (Tau-Weiss) (**Figure 1** **and** **Table 1**). End systolic pressure, end systolic volume and stroke volume in caPI3K CLP mice were maintained at levels significantly higher than the WT CLP mice, but they were significantly different from the sham controls.

**Table 1:** Cardiac myocyte specific expression of PI3K p110α significantly attenuated cardiac dysfunction in caPI3K mice with CLP induced polymicrobial sepsis.

Hemodynamic parameter	Wild Type Sham	Wild Type CLP	caPI3K Sham	caPI3K CLP
Heart rate (beats/min)	419.7±47.4	280.2±50.6^1^	441.8±82.8	552.3±7.8^2,3^
End systolic pressure (mmHg)	93.6±11.16	77.6±17.21	96.9±7.8	104.1±15.1^2^
End diastolic pressure (mmHg)	3.3±0.8	2.4±0.3	4.2±1.8	3.4±1.4
End systolic volume (µl)	37.0±7.6	28.9±3.8	38.9±3.5	28.5±1.9^3^
End diastolic volume (µl)	66.4±3.4	37.9±2.9^1^	69.0±3.4	48.8±3.8^2,3^
Stroke volume (µl)	32.3±3.3	10.1±3.0^1^	34.4±3.2	22.8±9.7^2,3^
Emax (mmHg/µl)	19.1±6.4	7.8±1.7^1^	12.7±4.6	15.3±2.8^2^
Tau-Weiss (ms)	6.4±1.3	12.0±2.5^1^	7.2±0.9	7.2±2.7^2^

Data are presented as mean ± SD.

1 p<0.05 vs wild type sham group.

2 p<0.05 vs wild type CLP group.

3 p<0.05 vs caPI3K sham group.

N = 5–6/group.

Echocardiographic analysis confirmed that caPI3K Tg mice showed attenuation of cardiac function in response to CLP sepsis (**Table 2**). Left ventricular end systolic diameter (LVESD) was significantly increased, while %FS and ejection fraction (%EF) were significantly decreased in WT CLP mice, when compared to WT sham and caPI3K CLP groups (**Table 2**). Interestingly, LVEDD did not change in response to CLP sepsis, but in the caPI3K-CLP group LVEDD was significantly lower than either the caPI3K-sham or WT-CLP groups (**Table 2**). There was no significant difference in LVESD, %FS and EF between caPI3K-sham and caPI3K-CLP groups.

**Table 2:** Echocardiographic analysis indicates that caPI3K mice maintain cardiac function in response to CLP sepsis.

Parameters	Wild Type Sham (n = 5)	Wild Type CLP (n = 5)	caPI3K Sham (n = 6)	caPI3K CLP (n = 6)
LVESD (mm)^1^	2.16±0.19	2.78±0.14^3^	2.36±0.3	2.03±0.22^4,5^
LVEDD (mm)^2^	3.65±0.14	3.56±0.12	3.67±0.25	3.18±0.27^4^,^5^
% fractional shortening	40.83±2.9	21.99±3.82^3^	35.78±5.80	36.12±5.12^4^
Ejection fraction	72.30±3.65	44.90±3.64^3^	65.56±7.78	66.78±6.45^4^

Data are presented as mean ± SD.

1 left ventricular end systolic diameter.

2 left ventricular end diastolic diameter.

3 p<0.05 vs wild type sham group.

4 p<0.05 vs wild type CLP group.

5 p<0.05 vs caPI3K sham group.

N = 5–6/group.

### Cardiac Specific Expression of Constitutively Active PI3K p110α Results in Increased Survival Outcome in CLP Sepsis

**Figure 2:**
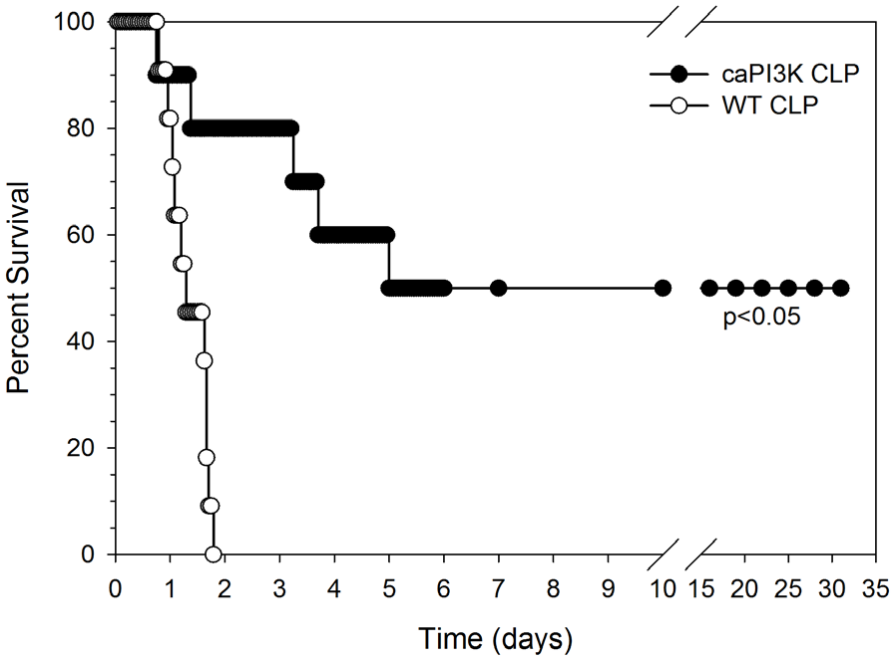
Cardiac myocyte specific expression of PI3K p110α significantly improves survival outcome in CLP-induced sepsis/septic shock. caPI3K transgenic mice (N = 10) and age-matched wild type mice (N = 11) were subjected to CLP at time 0. The mice were followed for survival for up to 30 days.

To investigate whether preservation of cardiac function during sepsis/septic shock will alter survival outcome, we performed CLP to induce sepsis/septic shock in caPI3K (p110α) mice (n = 10). Age-matched wild type (WT) mice (n = 10) served as control. As shown in **Figure 2**, caPI3K mice were more resistant to CLP-induced mortality than WT mice. The median survival time in WT mice was ∼28 hrs and time to 100% mortality was 46 hrs. In striking contrast, 90% of the caPI3K mice were alive at ∼28 hrs and 80% of the caPI3K mice survived at 46 hrs after CLP (**Figure 2**). Of greater significance, 50% of the caPI3K mice went on to survive indefinitely (>30 days) after CLP.

### Constitutive Activation of Myocardial PI3K p110α Results in Increased Phosphorylation of Akt in the Myocardium, but not Other Tissues

**Figure 3:**
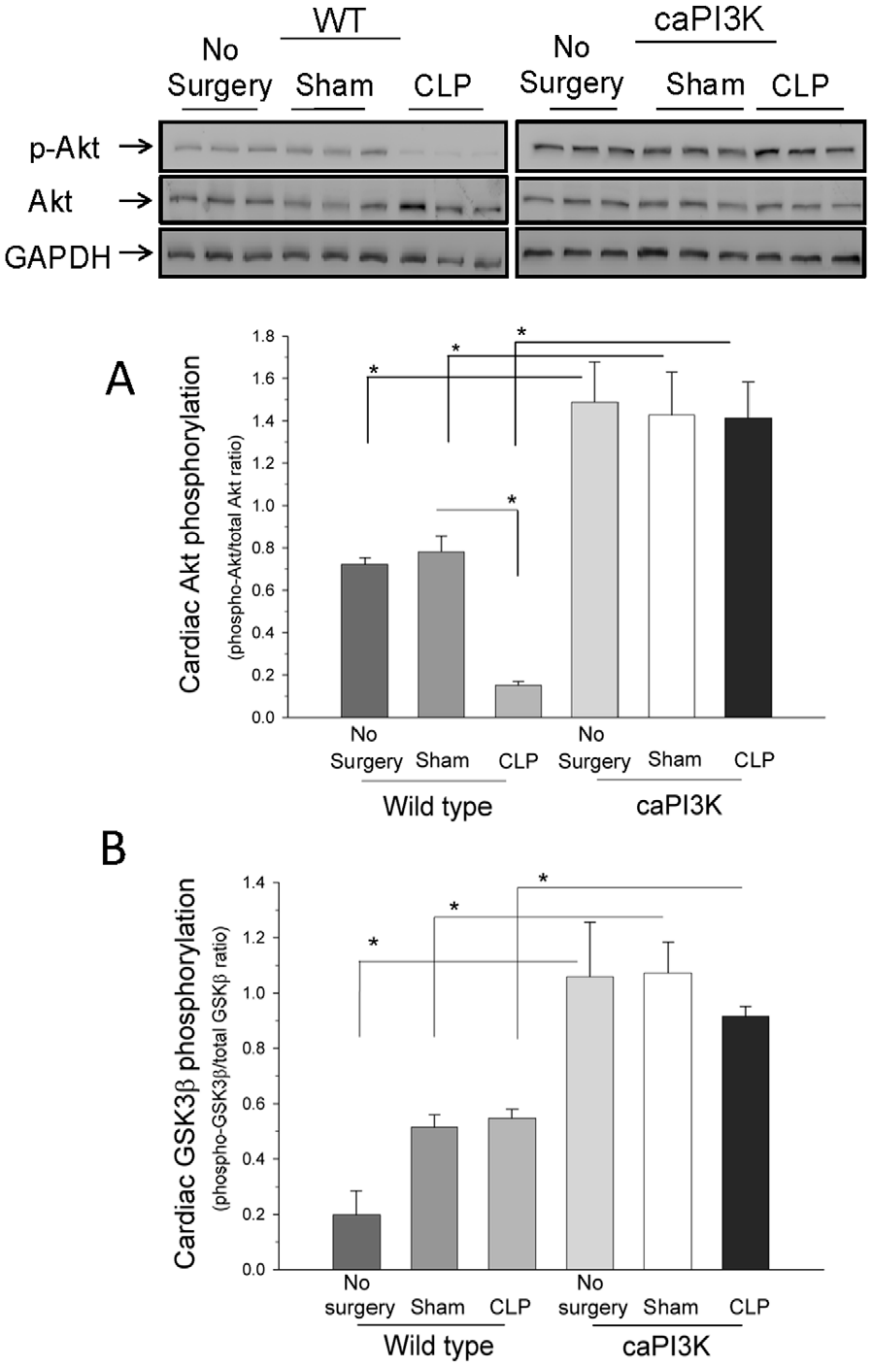
Constitutive activation of myocardial PI3K p110α results in increased phosphorylation of Akt and GSK3β in the myocardium. A . Akt phosphorylation is increased in the myocardium of caPI3K Tg mice, when compared to WT mice. Six hrs after CLP, the hearts were harvested and cellular proteins were examined for levels of phospho-Akt/Akt. *p<0.05 compared with indicated group. (n = 6/group). **B**. Expression of constitutively active PI3K p110α increases myocardial GSK3β phosphorylation in caPI3K mice. caPI3K mice and age-matched WT mice were subjected to CLP. Six hrs after CLP, the hearts were harvested and cellular proteins were examined for levels of phospho-GSK3β. *p<0.05 compared with indicated group. (n = 6/group).

Akt is a downstream target of PI3K [9]. Akt is activated by phosphorylation [9]. We examined the levels of myocardial Akt phosphorylation as well as total Akt levels in control and septic mice (**Figure 3A**). Phosphorylated Akt levels were increased in the myocardium of normal control, sham control and CLP mice expressing the p110α transgene, when compared to WT mice (**Figure 3A**). Interestingly, we did not see any significant difference in myocardial Akt phosphorylation in p110α transgenic mice in the presence or absence of sepsis, however, sepsis did result in a significant decrease in myocardial Akt phosphorylation in wild type mice. We also examined Akt phosphorylation in the liver, lung and spleen of caPI3K Tg mice in the presence and absence of sepsis. We did not find any differences in Akt phosphorylation in organs other than the heart in the presence or absence of sepsis (data not shown). These data indicate that constitutive activation of myocardial PI3K p110α activates Akt in the myocardium, but it does not appear to activate Akt in extra-cardiac tissues. This is consistent with previous observations indicating that the effect of the transgene is limited to the heart [20].

### Constitutive Activation of Myocardial PI3K p110α Results in Increased Phosphorylation of GSK3β in the Myocardium, but not Other Tissues

Activated Akt phosphorylates several downstream targets of the PI3K pathway including glycogen synthase kinase-3β (GSK3β) [32], [33]. GSK3β is a crucial regulator of many cellular functions, including cell survival and apoptosis [32]. GSK3β is a constitutively active enzyme that is inactivated by Akt via phosphorylation of serine 9 [32]. The levels of phosphorylated GSK3β were increased in the myocardium of normal control, sham control and CLP mice expressing the p110α transgene when compared to WT mice (**Figure 3B**). These data indicate that constitutive activation of myocardial PI3K p110α inactivates GSK3β in the myocardium. Interestingly, we did not see any significant difference in myocardial GSK3β phosphorylation in p110α transgenic mice in the presence or absence of sepsis. We also examined the phosphorylation of GSK3β in tissues other than the heart. There were no differences in GSK3β phosphorylation in tissues other than the heart, *i.e.* liver, lung and spleen (data not shown), in the presence or absence of sepsis. These data indicate that constitutive activation of myocardial PI3K p110α inactivates GSK3β in the myocardium in the presence or absence of sepsis, but it does not appear to inactivate GSK3β in extra-cardiac tissues.

### Serum Cytokine/chemokine Levels are Maintained at Normal Levels in Septic Mice with Cardiac Specific Expression of PI3K p110α

**Figure 4:**
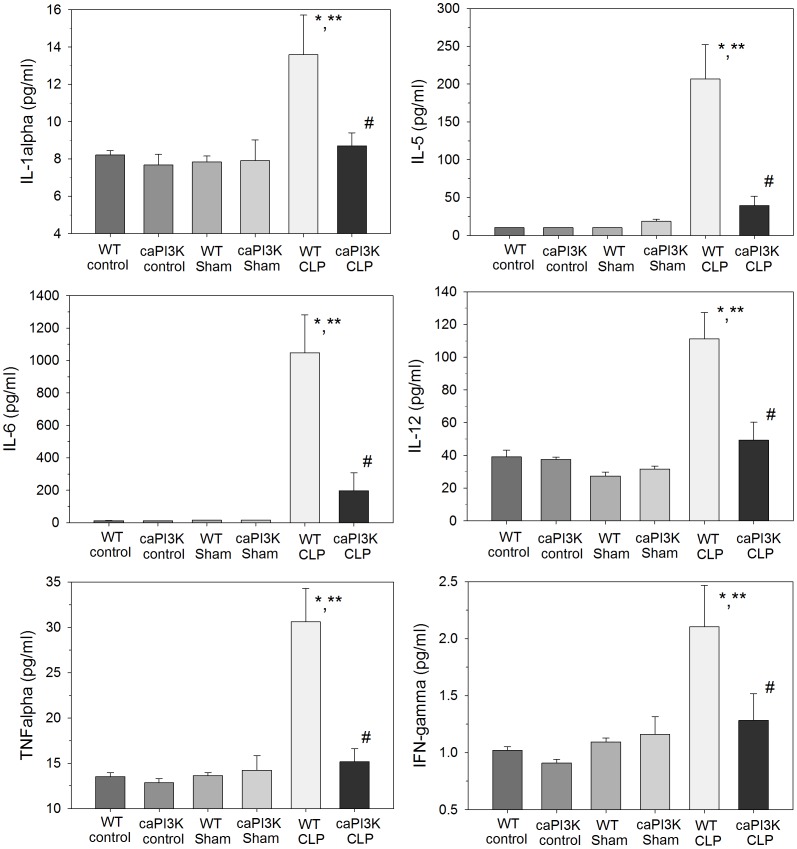
Cardiac specific expression of PI3K p110α attenuates serum cytokine expression during CLP sepsis. caPI3K Tg mice and age-matched WT mice were subjected to CLP. Sixteen hours after CLP the mice were euthanized, serum was harvested and analyzed for cytokine/chemokine levels. *p<0.05 vs WT control; **p<0.05 vs WT sham surgery; #p<0.05 vs WT CLP. (n = 4–6/group).

We examined serum cytokine/chemokine levels at 16 hrs after induction of CLP sepsis (**Figs. 4** **and** **5**). As previously reported, CLP sepsis resulted in a significant increase in serum cytokine levels at 16 hrs post-surgery (**Figs. 4** **and** **5**). In striking contrast, expression of cardiac specific PI3K p110α had a dramatic effect on CLP induced serum cytokine levels. Serum cytokine/chemokine levels were maintained at normal levels in caPI3K CLP mice (**Figs. 4** **and** **5**). Specifically, serum IL-1α, IL-5, IL-6, IL-12, IL-17, TNFα, IFN-γ, IP-10, MCP-1, MIG and MIP-1α levels in caPI3K Tg mice were not significantly different from control and sham mice (**Figs. 4** **and** **5**). These data indicate that septic caPI3K Tg mice show a predominantly normal inflammatory profile, when compared to WT CLP mice. No significant changes were observed in IL-1β, IL-2, IL-4, IL-13, GM-CSF, FGF_basic_ or GM-CSF in caPI3K Tg mice in the presence or absence of sepsis (data not shown).

**Figure 5:**
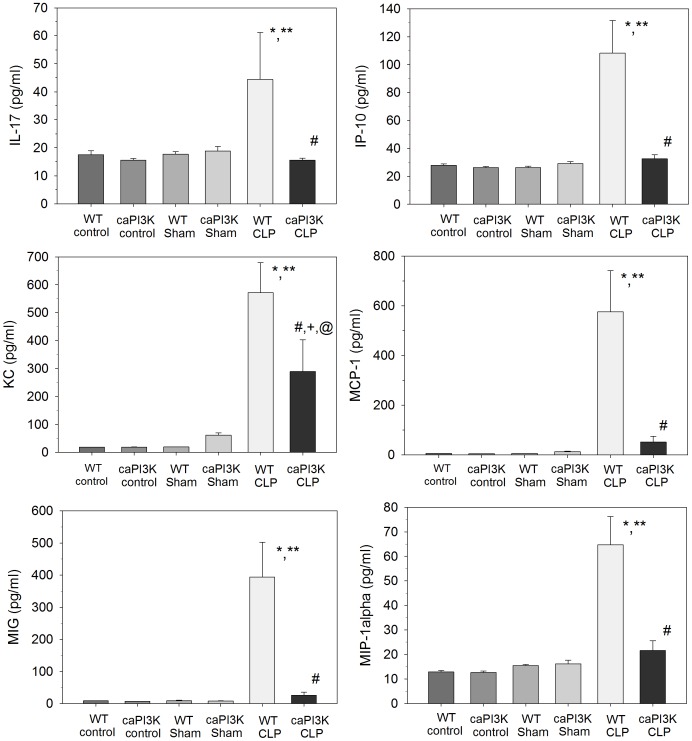
Cardiac specific expression of PI3K p110α attenuates serum cytokine expression during CLP sepsis. caPI3K Tg mice and age-matched WT mice were subjected to CLP. Sixteen hours after CLP the mice were euthanized, serum was harvested and analyzed for cytokine/chemokine levels. *p<0.05 vs WT control; **p<0.05 vs WT sham surgery; #p<0.05 vs WT CLP; ^+^p<0.05 vs caPI3K control; ^@^p<0.05 vs caPI3K sham surgery control. (n = 4–6/group).

### Attenuation of Serum KC Levels in Septic Mice with Cardiac Specific Expression of PI3K p110α

KC is the murine equivalent of human IL-8, a neutrophil chemoattractant and activator [34]. We observed that CLP in WT mice significantly increased serum KC levels (**Fig. 5**). However, septic mice with cardiac specific expression of PI3K p110α showed attenuation (↓49.4%) of serum KC levels. Despite this attenuation, serum KC levels in the caPI3K mice were significantly elevated when compared to control and sham control mice (**Fig. 5**).

### Attenuation of CLP Induced NFкB Activation in caPI3K Mice

**Figure 6:**
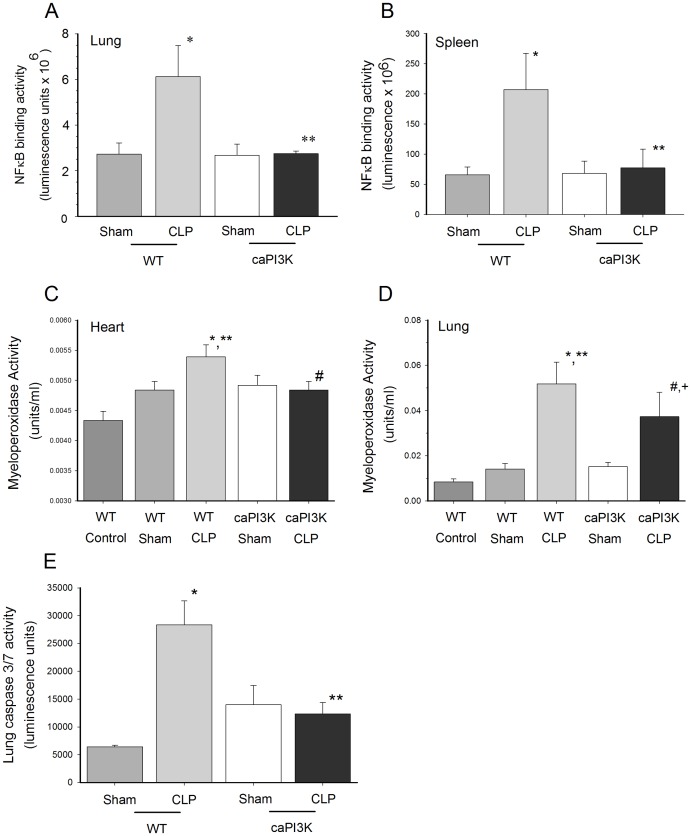
Cardiomyocyte expression of the p110α transgene attenuated tissue NFкB activity in response to CLP sepsis. Lung (**A**) and spleen (**B**) NFкB activity are shown at the top. caPI3K mice and age-matched WT mice were subjected to CLP. Lung tissue was harvested 16 hrs after CLP. *p<0.05 vs WT control; **p<0.05 vs WT CLP group. (n = 4/group). Cardiac specific expression of PI3K p110α attenuated myeloperoxidase activity in the heart (**C**) and lung (**D**) in response to sepsis. caPI3K mice and age-matched WT mice were subjected to CLP. Heart and lung tissue was harvested 16 hrs after CLP. (n = 4–6/group). ^*^p<0.05 vs WT; ^**^p<0.05 vs caPI3k control; ^#^p<0.05 vs WT CLP; ^+^p<0.05 vs caPI3k sham. **E.** Lung caspase 3/7 activity was increased in wild type, but not caPI3K Tg, mice in response to CLP sepsis. caPI3K mice and age-matched WT mice were subjected to CLP. Lung tissue was harvested 16 hrs after CLP. (n = 4–6/group). *p<0.05 vs WT control; **p<0.05 vs WT CLP group.

Sepsis induced activation of tissue NFкB activity is associated with increased morbidity and mortality [21]. NFкB is also known to play a pivotal role in the regulation of cytokine/chemokine expression [35]. The data in **Figures 4** **and** **5** indicate that caPI3K mice do not show a pro-inflammatory phenotype. This observation prompted us to examine organ NFкB activity in caPI3K mice in response to CLP sepsis. As expected, WT mice showed a significant increase in lung and spleen NFкB activity in response to sepsis (**Figure 6A and 6B**). However, lung and spleen tissue from mice expressing the p110α transgene did not show nuclear translocation of NFкB components in response to sepsis (**Figure 6A and B**). Surprisingly, we did not see any change in cardiac NFкB activity in the presence or absence of sepsis (data not shown). Thus, the NFкB confirms and extends the cytokine/chemokine data which indicates that caPI3K mice show a predominantly normal phenotype with respect to inflammatory mediators.

### Attenuation of Tissue MPO Activity in Septic caPI3K Mice

Neutrophil accumulation and degranulation in organs during CLP sepsis is thought to mediate some of the pathophysiology of this devastating disease [36]. Even though caPI3K mice showed attenuation of serum KC levels, a neutrophil chemo-attractant, in response to sepsis, it was noted that KC levels were higher in septic caPI3K mice than in control or sham caPI3k mice (**Fig. 5**). To evaluate the effect of transgenic p110α on neutrophils in sepsis, we examined tissue MPO activity as an indicator of tissue neutrophil infiltration and accumulation [37]. Tissue MPO activity was increased in the heart and lung of WT CLP mice at 16 hrs after surgery (**Fig. 6C and D**). MPO activity in the heart of caPI3K mice was not significantly elevated in response to sepsis and it was significantly lower than the MPO levels in the WT CLP myocardium, indicating that expression of the p110α transgene prevented neutrophil accumulation in the heart. MPO activity was also significantly elevated in the lungs of WT mice at 16 hrs after CLP, indicating neutrophil accumulation (**Figure 6D**). Lung MPO activity was 27.4% lower (p<0.05) in septic caPI3K mice, when compared to the WT CLP mice. However, lung MPO activity in septic caPI3K Tg mice was increased (p<0.05) when compared to caPI3K Tg sham control mice (**Fig. 6D**), indicating that myocardial expression of the p110α transgene can attenuate, but did not completely prevent, neutrophil accumulation in the lung.

### Cardiac Specific Expression of PI3k p110α Prevented Activation of Tissue Caspase 3/7 and 8 in CLP Mice

Apoptosis is thought to play a significant role in pathophysiology of sepsis [38]. PI3K signaling has been reported to be a pro-survival, anti-apoptotic pathway [11], [12]. We examined caspase 3/7 and 8 as indicators of the intrinsic and extrinsic apoptotic signaling pathways. CLP significantly increased lung caspase 3/7 activity at 16 hrs (**Figure 6E**). However, caPI3K Tg mice showed no change in caspase 3/7 activity in response to CLP (**Figure 6E**). We did not detect any significant difference in lung caspase 8 activity (data not shown). We did not detect changes in heart or liver apoptosis in response to CLP.

### Cardiac Specific Expression of the p110α Transgene did not Alter Splenocyte Apoptosis in Response to CLP Sepsis

**Figure 7:**
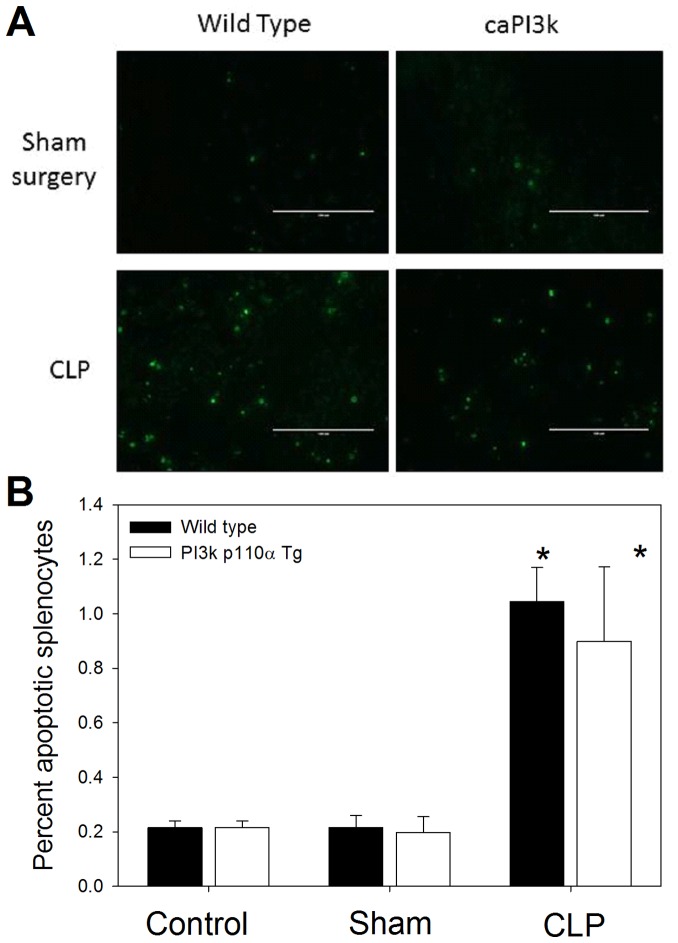
Expression of cardiac specific PI3K p110α did not significantly alter splenocyte apoptosis in response to CLP sepsis. Splenocytes were harvested 16 h after induction of CLP sepsis. Splenocyte apoptosis was assessed by TUNEL staining (A) and flow cytometry (B). *p<0.05 vs control or sham treatment. N = 4–6/group.

We [11] and others [12] have reported that CLP sepsis induces splenocyte apoptosis. Activation of PI3K/Akt dependent signaling has been reported to attenuate splenocyte apoptosis in sepsis [11], [12]. In this study we examined splenocyte apoptosis at 16 hrs post-CLP. We examined apoptosis by TUNEL staining of splenic tissue (**Figure 7A**) and flow cytometry of isolated splenocytes (**Figure 7B**). As expected, CLP sepsis significantly increased splenocyte apoptosis in WT mice. Mice expressing the cardiac specific p110α transgene showed comparable levels of splenocyte apoptosis with both methods, indicating that cardiac specific expression of PI3K p110α did not alter sepsis induced splenocyte apoptosis (**Figure 7A and B**).

### Cardiac Specific Expression of PI3K p110α Inhibits Sepsis Induced Release of HMGB-1 from the Nucleus to the Cytoplasm in Cardiac Myocytes

**Figure 8:**
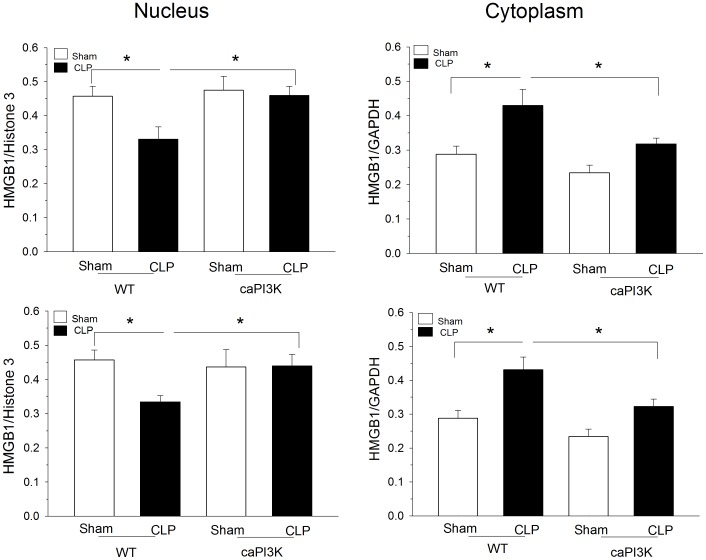
Cardiac specific expression of PI3K p110α inhibits HMGB-1 release from the nucleus to the cytoplasm in cardiac myocytes. Nuclear and cytoplasmic HMGB-1 levels are shown at 6 (top) and 12 hrs (bottom) after CLP. caPI3K Tg mice and age-matched WT mice were subjected to CLP. Six and twelve hours after CLP the mice were euthanized and nuclear and cytoplasmic protein was harvested. HMGB-1 was assessed by Western blot. *p<0.05 WT CLP vs WT sham control or caPI3k CLP. N = 4–6/group.

HMGB-1 is a late mediator of septic sequelae that is released from the nucleus into the cytosol and systemic circulation in response to various stimuli including sepsis [39]–[42]. We examined the effect of myocardial caPI3K on release of nuclear HMGB-1 in cardiac myocytes in response to CLP sepsis (**Figure 8**). We examined two time intervals, six (top) and twelve (bottom) hours after CLP. As expected, WT CLP mice showed a loss of nuclear HMGB-1 and an increase in cytoplasmic levels at both time intervals (**Figure 8**). In striking contrast, caPI3K mice, subjected to CLP sepsis, showed no significant translocation of HMGB-1 from the nucleus to the cytoplasm.

## Discussion

In this study we found that constitutive activation of myocardial PI3K p110α dramatically attenuates the cardiac dysfunction associated with polymicrobial sepsis. We critically examined a broad range of cardiac functional parameters in order to better understand the role of myocardial PI3K p110α activation in the attenuation of cardiac dysfunction during sepsis. As shown above, cardiac output was dramatically reduced in wild type mice in response to sepsis, suggesting inadequate perfusion of vital organs as well as inadequate blood return to the left ventricle. However, septic caPI3K mice showed higher heart rates, although a smaller stroke volume was observed in these mice. The decrement in stroke volume was offset by the higher heart rate in septic caPI3K mice resulting in cardiac output that was maintained at normal levels. The data also indicate that blood return to the heart was maintained at or near normal levels in septic caPI3K mice. In response to sepsis, wild type mice showed significant decreases in left ventricular contractility. In contrast, constitutive activation of PI3K p110α in cardiac myocytes resulted in left ventricular contractility that was maintained at normal levels during sepsis. We also observed that myocardial activation of PI3K p110α maintained normal compliance of the left ventricle during sepsis, while ventricular wall stiffness was markedly increased in septic wild type mice. It was also noted that in contrast to wild type mice, constitutive activation of cardiomyocyte PI3K p110α resulted in left ventricular elastance and compliance that was maintained at normal levels, thus preserving normal left ventricular function during sepsis. When taken together, these data indicate that sepsis negatively impacts a broad range of left ventricular functional parameters. However, myocardial activation of PI3K p110α prevents or significantly attenuates many of the deleterious effects of polymicrobial sepsis on left ventricular function. The overall maintenance of cardiac function in caPI3K mice strongly correlates with decreased morbidity, attenuation of the pro-inflammatory phenotype and improved survival outcome in sepsis.

Akt (aka = protein kinase B) is an important downstream target of PI3K [9], [33]. In this study we observed that sepsis attenuated myocardial Akt phosphorylation in wild type mice, but not caPI3k Tg mice. Furthermore, Akt phosphorylation (activation) was only increased in the myocardium of caPI3k Tg mice. Therefore, the effect of the p110α transgene did not significantly influence Akt phosphorylation at extra-cardiac sites. This is consistent with previous reports using the PI3K transgenic mice [20]. Based on these data it is reasonable to conclude that the effect of the transgene was primarily observed in the heart and that the systemic effect is due to an overall maintenance of left ventricular cardiac function during sepsis. Activated Akt phosphorylates several downstream targets of the PI3K pathway including GSK3β [32], [33]. GSK3β is a crucial regulator of many cellular functions, including cell survival and apoptosis [32]. GSK3β is a constitutively active enzyme that is inactivated by Akt via phosphorylation of serine 9 [32]. We found that constitutive activation of myocardial PI3K p110α increased the levels of phosphorylated GSK3β, indicating inhibition of this enzyme in the myocardium. Inhibition of myocardial GSK3β positively correlated with maintenance of cardiovascular function and improved survival outcome in CLP sepsis.

The precise role of circulating cytokines in the pathophysiology of sepsis/septic shock is controversial and there is no definitive cause-and-effect relationship between systemic cytokine levels and survival outcome in sepsis [43]. Nevertheless, an examination of circulating cytokines can provide valuable information on the inflammatory and immunologic phenotype in response to CLP sepsis [11]. Our data clearly show that cardiac specific expression of PI3K p110α prevents cytokine/chemokine over expression in response to sepsis. Indeed, serum cytokine/chemokines in caPI3K mice remained at control levels in response to sepsis. Therefore, caPI3K did not show a pro-inflammatory phenotype in response to sepsis. These data also indicate that we did not see a shift from the Th1 to the Th2 phenotype that is frequently associated with fulminating sepsis [44]. These results also indicate that constitutive activation of p110α inhibited myocardial translocation of HMGB1. HGMB1 is thought to play a role in mediating organ damage in severe sepsis [41], [40]. It is reasonable to speculate that the effects of myocardial caPI3K p110α on HMGB-1 may contribute to the protective mechanism. An intriguing aspect of this investigation is that the PI3k p110α transgene was only expressed in cardiomyocytes and is not expressed in immune competent cells, such as macrophages, neutrophils and lymphocytes. Based on the current model of sepsis, it would have been reasonable to expect that macrophages and other immunocytes from caPI3K mice would respond normally to CLP sepsis by releasing pro-inflammatory mediators with a subsequent shift to a pro-inflammatory phenotype. This should have occurred irrespective of the PI3K p110α transgene effect in the heart. However, our data clearly indicate that this did not occur. Therefore, our data suggest that the current dogma regarding how immune competent cells respond during sepsis may not be entirely accurate. Specifically, our data suggest that maintaining left ventricular function during sepsis may improve perfusion of the peripheral vasculature and thereby attenuate the systemic inflammatory response.

Activation of tissue NFкB activity is associated with increased morbidity and mortality in CLP sepsis [21], [45]. Furthermore, we have reported that blunting tissue NFкB nuclear translocation will reduce morbidity and enhance survival outcome in CLP sepsis [45]. NFкB is also known to play a pivotal role in the regulation of cytokine/chemokine expression during septic sequelae [45]. As expected, WT mice showed a significant increase in lung NFкB activity in response to sepsis, which correlated with a strong pro-inflammatory response and rapid mortality. However, lung and splenic tissue from mice expressing the p110α transgene did not show nuclear translocation of NFкB components in response to sepsis. Not surprisingly, the NFкB data confirms and extends the cytokine/chemokine data, indicating that caPI3K mice show a predominantly normal inflammatory phenotype in response to sepsis.

Tissue neutrophil infiltration, adhesion and degranulation are thought to play a prominent role in tissue damage during sepsis [46]. KC is the murine equivalent of human IL-8 [34]. Like IL-8, KC plays a significant role in neutrophil chemotaxis and activation [34]. We found that serum KC levels in septic caPI3K Tg mice were increased relative to the controls, but KC levels were significantly lower in septic caPI3K Tg mice when compared to WT CLP mice. We examined tissue myeloperoxidase (MPO) levels as an index of tissue neutrophil infiltration and sequestration. Cardiomyocyte p110α expression resulted in a normalization of MPO levels in the heart of CLP mice, indicating that transgene expression prevented neutrophil sequestration in the heart during sepsis. In the lung of caPI3K CLP mice we observed a pattern of MPO activity that closely mirrored the serum KC response to sepsis. Specifically, the expression of the p110α transgene significantly attenuated sepsis induced neutrophil infiltration into the lung. However, the increase in lung MPO, i.e. neutrophil levels, did not result in increased apoptosis in the lung. Indeed, caspase 3/7 and 8 activity were at control levels in the heart and lung of caPI3k mice in response to sepsis. Interestingly, splenocyte apoptosis, which is a hallmark of CLP sepsis, was increased in both WT and caPI3K mice. This suggests that the beneficial effect of cardiac specific expression of p110α does not prevent sepsis induced splenocyte apoptosis. We have previously reported that systemic activation of PI3K with a pharmacologic agent decreased morbidity, prevented cardiac dysfunction and increased long term survival in CLP sepsis in a manner very similar to that observed in mice expressing the p110α transgene [11], [18]. However, there was one significant difference. In those studies we found that systemic activation of PI3K prevented sepsis induced splenocyte apoptosis, while at the same time maintaining cardiac function and enhancing survival outcome. When taken together, these data suggest that CLP sepsis does not mediate splenocyte apoptosis via cardiac dysfunction, rather it is mediated through an alternative mechanism. These data also suggest that transient and systemic activation of PI3K/Akt [11] may be more beneficial in sepsis than cardiac specific PI3K p110α activation.

Our data indicate that activation of a single isoform of PI3K (p110α), in a single cell type (the cardiac myocyte), in a single organ (heart), can have a profound effect on the morbidity and mortality associated with fulminating sepsis. The precise mechanism(s) by which myocardial PI3K p110α activation induces such dramatic effects at the systemic level are not entirely clear. It is reasonable to speculate that maintaining left ventricular function in sepsis/septic shock would have systemic benefits due to the maintenance of peripheral vascular perfusion, blood pressure and related issues. To the best of our knowledge, this is the first demonstration of a causal relationship between PI3K/p110α, the cardiac myocyte, cardiac function and survival outcome in sepsis. These data have practical implications for the management of sepsis, sepsis syndrome and septic shock. We have reported that transient and systemic activation of PI3K by a pharmacologic agent results in a maintenance of cardiovascular function and decreased morbidity and mortality in sepsis [11], [18]. The decreased morbidity and mortality reported in that study is consistent with the data observed in the current study, which employed tissue specific genetic up regulation of PI3K p110α. Therefore, transient pharmacologic up regulation of PI3K/Akt signaling pathways may be beneficial in the prevention and/or management of sepsis and septic sequelae.
